# Use of PSMA PET/CT to detect prostate cancer metastatic to a preexisting thyroid nodule

**DOI:** 10.1038/s41698-024-00619-5

**Published:** 2024-06-15

**Authors:** Cameron Chalker, Burçak Yilmaz, Kristin Trone, Genevieve Parecki, Athena Chen, James Y. Lim, Nadine Mallak, Alexandra O. Sokolova

**Affiliations:** 1 Department of Medical Oncology, Oregon Health & Science University (OHSU), 3181 S.W. Sam Jackson Park Road, Portland, OR 97239 USA; 2 Department of Diagnostic Radiology, OHSU, 3181 S.W. Sam Jackson Park Road, Portland, OR 97239 USA; 3 Department of General Surgery, OHSU, 3181 S.W. Sam Jackson Park Road, Portland, OR 97239 USA; 4 Department of Anatomic and Clinical Pathology, OHSU, 3181 S.W. Sam Jackson Park Road, Portland, OR 97239 USA; 5 Department of Surgical Oncology, OHSU, 3181 S.W. Sam Jackson Park Road, Portland, OR 97239 USA

**Keywords:** Cancer imaging, Cancer genomics, Prostate cancer

## Abstract

Prostate cancer (PCa) seldom metastasizes to the thyroid gland, and only a limited number of cases are documented in the literature. The application of a relatively recent and highly sensitive imaging technique, prostate-specific membrane antigen (PSMA) positron emission tomography—computed tomography (PET/CT), has enhanced the identification of metastatic disease. Nevertheless, as PSMA is expressed in various tissue types, the clinical importance of a PSMA-avid thyroid lesion remains largely uncertain. A minor, yet noteworthy, percentage of these lesions are ultimately determined to be malignant. Here we describe the case of a 70-year-old man with a past medical history of Lynch syndrome who presented to an outpatient oncologic clinic for management of very high risk localized PCa. He developed metastatic recurrence and his disease progressed through several lines of therapy, including immunotherapy and targeted treatments. He was found to have a new, intense PSMA uptake in an existing, previously benign thyroid nodule. Sonographic evaluation revealed changing morphology despite grossly stable size. Repeat biopsy confirmed the unusual finding of PCa metastasis to a known thyroid nodule. The shift in PSMA avidity played a pivotal role in discerning this metastatic deposit. There is a potential risk that such lesions may be inadequately acknowledged. The impact of the patient’s Lynch syndrome on this presentation remains uncertain.

## Introduction

Thyroid nodules are increasingly common—found in up to 68% percent of the population^1^. Fewer than 10% of all thyroid nodules are malignant and most of these neoplasms arise from the thyroid itself^2^. The data are varied, but in clinical series, about 1.9% of all malignancies found in the thyroid are reported to arise from a primary cancer outside of the thyroid^3^. Solid tumors originating from the kidney, lung, breast, and gastrointestinal (GI) tract are the prevalent sources of metastases to the thyroid gland^4–9^; additionally, cases of primary lymphoma have been documented^10^. Far less common is metastasis of prostate cancer (PCa) to the thyroid gland, with only a handful of cases reported^11–16^.

Prostate-specific membrane antigen (PSMA) is a type II transmembrane glycoprotein which is upregulated and strongly expressed in PCa cells, especially in hormone refractory PCa and its metastases^17,18^. PSMA expression has also been demonstrated in normal tissue, such as the renal tubules and in the neovasculature of non-prostate malignancies^19^. In recent years, surveillance and staging for PCa has begun to include PSMA positron emission tomography—computed tomography (PET/CT), a modality with increased sensitivity relative to conventional imaging^20–23^. Several PSMA PET agents are now available. The most commonly used—and reported in the literature—is 68-Gallium (68Ga)-PSMA-11, although the use of 2-(3-{1-Carboxy-5-[(6-[18F]fluoro-pyridine-3-carbonyl)-amino]-pentyl}-ureido)-pentanedioic acid (18F-DCFPyL) has steadily increased since its Food and Drug Administration (FDA) approval in 2021^18,24^. As the use of PSMA PET becomes more widespread, new data is emerging regarding the likelihood and implications of uptake at sites considered unusual for prostate cancer metastatic involvement^25,26^. The significance of PSMA PET-avid lesions in the thyroid gland is still largely unknown. Recent meta-analyses suggest that 6–26% of these thyroid gland incidentalomas could be malignant^27,28^.

Here, we describe the case of a patient with metastatic prostate cancer who was found to have metastasis not just to the thyroid gland but also to a preexisting thyroid nodule, a finding that has not yet been described in the literature.

## Results

### Case description

**Fig. 1:**
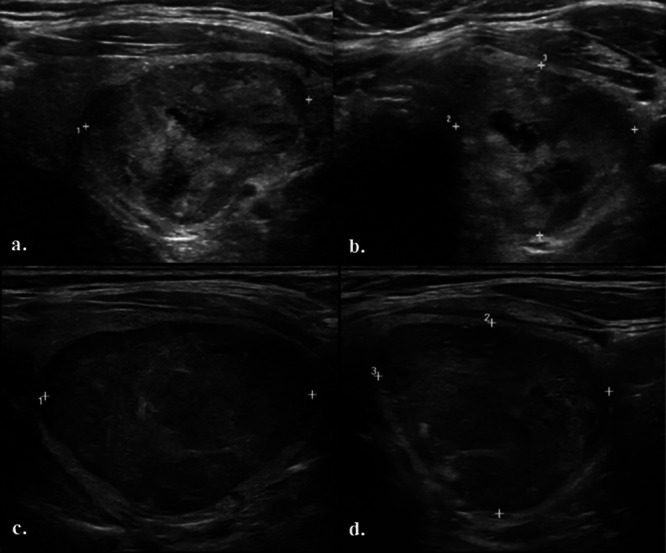
Thyroid ultrasonography. Previous ultrasonography from Spring 2015: **a** Sagittal and (**b**) axial images revealed 3.4 × 2.7 × 2.6 cm heterogeneous solid nodule with small cystic components in left thyroid gland. New ultrasonography from September 2023: **c** Sagittal and (**d**) axial images demonstrated mild increase in size of the nodule, now diffusely solid and hypoechoic measuring 4 × 3 × 3.5 cm.

This is a 70-year-old man with a history of Lynch syndrome with a germline mutation in *PMS2* and castrate resistant PCa. He initially presented in 2016 with stage IIIc (T2bN0M0) disease. Gleason score at diagnosis was 5 + 5 = 10; prostate-specific antigen (PSA) was 166 ng/dl. There was no evidence of metastases on conventional imaging. He was noted to have a longstanding history of a left inferior thyroid gland nodule, first identified with ultrasonography (US) (Fig. 1) 2 years prior to his PCa diagnosis; radiographic features were reassuring at that time and fine-needle aspirate (FNA) was performed in 2015 and returned as Bethesda II, benign. Next-generation sequencing (NGS) of the original prostate tumor biopsy identified the presence of a somatic *BRCA2* mutation, the patient’s germline *PMS2* mutation, and 13.1 mutations/Mb tumor mutational burden (TMB). Interestingly, despite Lynch syndrome and high TMB, the original prostate tumor was microsatellite stable (MSS). He received definitive radiation to the prostate and seminal vesicles, along with 2 years of leuprolide and adjuvant docetaxel for six cycles. Eighteen months after completion of chemotherapy, he experienced metastatic recurrence and was initiated on abiraterone, followed by rucaparib at progression. After progression on rucaparib, he remained on leuprolide monotherapy for approximately 3 years with slowly rising PSA. In this interval time, he underwent both conventional imaging and PSMA PET/CT on several occasions; repeat thyroid ultrasonography was not performed.

In the spring of 2023, PSA doubling time significantly shortened and imaging showed new soft tissue metastases. A repeat biopsy of a metastatic deposit (left humerus) was performed. This lesion was noted to have high microsatellite instability (MSI-H) and a high TMB at 18 mutations/Mb. The previously-noted *BRCA2* mutation was not observed, however, mutations in *MSH3*, *MSH6*, *PMS2*, *CHEK2*, and *MEN1* were seen. Pembrolizumab was then prescribed given the patient’s history of Lynch syndrome and MSI-H/high TMB status. Unfortunately, his disease continued to progress (clinically and radiographically), and pembrolizumab was stopped after three cycles of therapy. PSA at this time was over 4000 ng/dl.

**Fig. 2:**
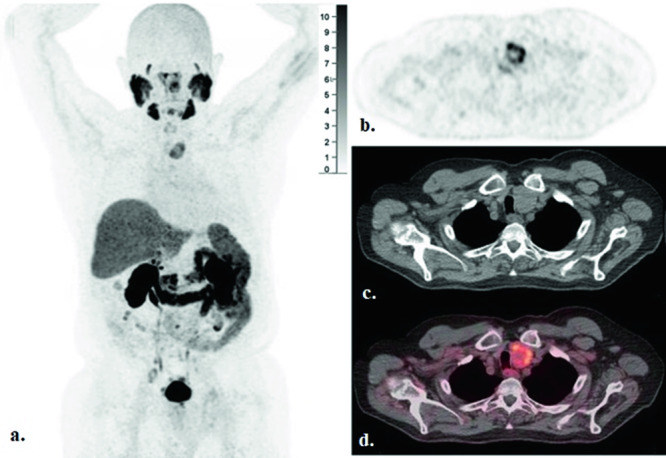
F-18 DCFPyL PET/CT imaging obtained before pembrolizumab start due to disease progression, with concurrent prostate-specific antigen level of 281 ng/ml in Spring 2023. **a** MIP, **b** Axial PET, **c** Axial CT, **d** Axial fusion PET/CT with PSMA uptake in a left thyroid nodule with dimensions of 3.6 × 3.0 cm (SUVmax: 8.2).

**Fig. 3:**
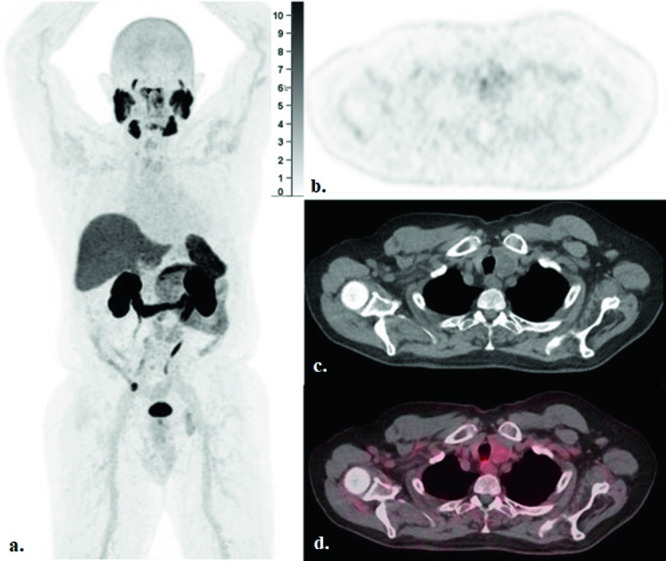
A prior F-18 DCFPyL PET/CT imaging obtained about 20 months since progression on rucaparib while patient was on ADT monotherapy, with concurrent prostate-specific antigen level of 25.4 ng/ml after systemic and radiation therapy in winter 2022. **a** Maximum INTENSITY PROJection (MIP), **b** Axial PET, **c** Axial CT and **d** Axial fused PET/CT images showed hypodense partially calcified 3.1 × 2.2 cm left thyroid nodule without notable radiotracer uptake (SUVmax: 3.6) (arrow).

**Fig. 4:**
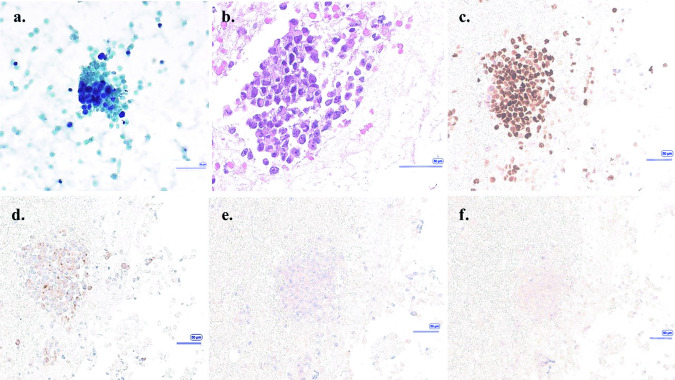
Thyroid fine-needle aspiration, Summer 2023: the cytology smear and cell block preparations show numerous hyperchromatic and atypical epithelial cells with high nuclear-to-cytoplasmic ratio, round to irregular nuclear contours, and prominent nucleoli. Abundant necrosis is present in the background. **a** Pap stain, 400x. **b** H&E stain on cell block preparation, 200x. Immunohistochemistry for NKX3.1, PSA, PAX8, and TTF1 confirm the diagnosis of metastatic prostatic adenocarcinoma. Tumor cells are positive for (**c**) NKX3.1 and (**d**) PSA, and negative for (**e**) PAX8 and (**f**) TTF1. Scale bar represents 50 µm in each image.

PSMA PET/CT obtained in the spring of 2023 prior to pembrolizumab initiation (Fig. 2) showed intense PSMA uptake in the left inferior thyroid gland at the site of the patient’s previously described nodule, with a maximum standardized uptake value (SUVmax) of 8.2. In contrast, no uptake (SUVmax = 3.6) was seen on prior PSMA PET/CT (Fig. 3) performed in the winter of 2022 while the patient was on ADT monotherapy, about 20 months after progression on rucaparib. This new uptake in the thyroid gland was highly suspicious, and a repeat thyroid US was performed (Fig. 1). On the new US, the left thyroid gland nodule demonstrated mild increase in size (from 3.4 to 4 cm in greatest dimension) with an associated change in morphology, now diffusely solid and hypoechoic concerning for malignancy. These findings were concerning for malignancy and a repeat FNA was obtained. The cytology smear and cell block preparations of the repeat FNA was notable for numerous atypical epithelial cells. These cells demonstrated a high nuclear-to-cytoplasmic ratio, hyperchromatic nuclei with round to irregular contours, and prominent nucleoli in a background of abundant necrosis. Immunohistochemical analysis revealed these atypical cells did not express general thyroid markers (PAX8 and TTF1), but rather expressed the prostate markers NKX3.1 and PSA, confirming the presence of prostate adenocarcinoma which had metastasized to the known thyroid gland nodule (Fig. 4). Repeated genomic analysis confirmed the presence of ongoing MSI-H and high TMB, as well as new *PTEN* and *TP53* loss. There were no mutations in homologous recombination repair pathway genes. As PCa was confirmed, surgical resection was deferred in favor of systemic therapy.

The patient subsequently received one cycle of cabazitaxel, followed by a cycle of cabazitaxel + carboplatin. Sadly, he was admitted to the hospital with septic shock and died shortly thereafter, after transitioning to comfort measures.

## Discussion

Metastases to the thyroid gland are uncommon in clinical practice, with the most common primary sites being kidney, breast, lung, and head & neck^4–9^. Thyroid metastases from prostate cancer have been very rarely described^11–16^. To our knowledge, this is the first known case of prostate cancer metastasis to an existing thyroid nodule.

There has been limited research on the appearance of thyroid gland incidentalomas with the use of different PET probes—mostly 18F-Fluorodeoxyglucose (FDG) and, more recently, 68Ga-PSMA-11^29,30^. The significance of PSMA uptake using 18F labeled PSMA agents, including 18F-DCFPyL, in thyroid gland incidentalomas is largely still emerging, particularly as PSMA is now known to be expressed in a number of non-prostate tissues, both malignant and benign^19^. A recent retrospective cohort found (Ga-PSMA-11, 18F-PSMA, or 18F-DCFPyL) PSMA-avid thyroid gland lesions in 1.1% of patients undergoing PSMA PET evaluation for PCa; of these, a small but clinically significant proportion (3 of 61) were malignant, with one differentiated thyroid carcinoma, one Hürthle cell carcinoma and one renal cell carcinoma metastasis^31^. A similar pattern was seen in a second meta-analysis of PSMA-avid incidentalomas; of the 23 lesions identified, 5 represented primary thyroid cancer while a sixth was a metastasis from renal cell carcinoma^32^. Studies have shown elevated PSMA expression in primary thyroid carcinomas in contrast to benign etiologies^30,33,34^. Increased PSMA avidity has also been observed in metastases from other solid malignancies, including renal cell carcinoma and lung adenocarcinoma^30,31,35^. Given the potential association with malignancy, the identification of an incidental PSMA-avid thyroid gland lesion should prompt further investigation.

A distinctive aspect of this case is the emergence of PSMA-avidity within our patient’s pre-existing thyroid gland nodule, previously non-avid. It was this change in avidity that raised suspicion for a malignant process, prompting US evaluation, subsequent biopsy, and impacting the patient’s treatment plan. Given the relative novelty of PSMA PET, it is possible that prostate gland metastases to the thyroid gland are more common than previously realized.

Also notable is the patient’s established diagnosis of Lynch syndrome, a well-described, autosomal dominant hereditary cancer syndrome that is caused by a germline mutation in DNA mismatch repair (MMR) such as *MLH1, MLH2, MSH6*, and *PMS2*. These genes, when functional, help to maintain genomic integrity by correcting small base pair substitution and insertion-deletion errors. Inactivation of both alleles is required for defective MMR function; patients with Lynch syndrome typically carry a germline mutation in one allele of an MMR gene, with loss of the second allele occurring somatically. This accumulation of DNA errors leads to a marked increase in cancer risk. Tumors that arise in patients with Lynch syndrome, therefore, commonly demonstrate MMR deficiency (dMMR). dMMR often correlates with the presence of a high TMB due to this increased capacity for somatic mutagenesis, although this is not uniformly true^36^, as was seen in our patient. The lifetime risk of prostate cancer in patients with Lynch syndrome may be as high as 30%^37^, more than twice that of non-carrier peers^38^; thus, prostate cancer is often regarded as a component of the Lynch syndrome tumor spectrum.

Patients with Lynch syndrome can experience remarkable response to checkpoint inhibition^39^. This is attributed to the increased immunogenicity of MSI-H and TMB-high tumors^39^ and a strong correlation between these genomic features and response to checkpoint inhibition has been demonstrated across multiple trials^40–43^. This ultimately resulted in the site-agnostic Food and Drug Administration (FDA) approval of pembrolizumab for patients with MSI-H or dMMR solid tumors^44,45^. Sadly, our patient’s disease continued to progress despite treatment with pembrolizumab and he passed away shortly thereafter. It is not clear if his history of Lynch syndrome contributed to this unusual presentation of PCa.

In conclusion, a change in PSMA-avidity at the site of a previously benign thyroid nodule ultimately identified the presence of a new metastatic deposit, a never-before described and clinically significant finding that may not have been appreciated without the use of this novel and highly sensitive imaging modality. Thyroid gland metastases in PCa have historically been considered very rare, however, it is possible that they are underrecognized. Whether the risk of thyroid gland metastases is increased in patients with Lynch syndrome is not yet known.

## Methods

Please see above for details of the patient’s presentation and clinical history. The diagnosis of Lynch syndrome was established prior to presentation. Somatic genomic analysis was performed via GeneTrails® 37-gene Comprehensive Solid Tumor Panel, a next-generation sequencing test that employs two amplicon-based libraries (DNA, RNA/cDNA). Microsatellite instability and estimated tumor mutational burden were also assessed using this panel. Testing was performed on three occasions: first, on the patient’s prostate biopsy, second, on a metastatic deposit obtained from the patient’s left humerus, and third, on the sample obtained from the thyroid nodule.

The patient’s clinical data was obtained under a protocol that was approved by the institutional review board of Oregon Health & Science University (FWA0000161), in compliance with the appropriate ethical regulations, including the Declaration of Helsinki. Additionally, a Request for Determination was submitted to OHSU IRB prior to submitting this case report for publication—it was determined that additional IRB oversight was not required for this publication. The patient provided written informed consent for publication.

Timeline:

**Table:** 

Date	Event
2015	Patient noted to have a 3.4 cm left inferior thyroid gland nodule (Fig. 1a, b). This was biopsied and was found to be benign.
2016	Patient diagnosed with stage IIIc (T2bN0M0) prostate cancer. Gleason score = 5 + 5 = 10. PSA at diagnosis = 166 ng/ml. Somatic tissue testing reveals a BRCA2 mutation, high TMB.
2017	Completes definitive radiation. Also receives adjuvant androgen deprivation therapy and adjuvant docetaxel chemotherapy.
Spring 2019	Noted to have metastatic recurrence. Initiated on abiraterone.
Fall 2019	Disease progression. Transitioned to rucaparib.
Spring 2021	Disease progression. Patient declined additional intervention, opting for ADT monotherapy with close PSA monitoring.
Winter 2021	PSMA PET/CT first obtained. This demonstrates regional nodal metastases without bone or visceral lesions. The thyroid nodule is described as “unchanged” from prior.
Winter 2022	Repeat PSMA PET/CT reveals increasing retroperitoneal adenopathy without bone or visceral involvement. The thyroid nodule is again noted to be stable (Fig. 3).
2022–2023	PSA steadily rises.
Spring 2023	Repeat PSMA PET/CT now demonstrates multiple foci of metastatic disease, including new and marked avidity in the left inferior thyroid gland nodule (SUV max 8.2) (Fig. 2). The lesion is described as being of “similar” size. This prompts biopsy of the left humerus, which confirms PCa, MSI-H, high TMB; previously-noted BRCA2 mutation no longer present. Repeat thyroid US is also performed (Fig. 1c, d). Relative to prior, the nodule of concern was found to be increased in size (4 cm), diffusely solid and hypoechoic, suggestive of malignancy.
Summer 2023	Initiated on pembrolizumab.
Summer 2023	Thyroid nodule remains of concern. FNA is performed and the lesion is confirmed to represent metastatic PCa (Fig. 4). Shortly thereafter, pembrolizumab is discontinued due to progressive disease and the patient is initiated on cabazitaxel.
Fall 2023	Carboplatin is added to treatment regimen
Fall 2023	Patient is admitted to the hospital with septic shock. He dies shortly thereafter, after transitioning to comfort measures.

### Reporting summary

Further information on research design is available in the Nature Research Reporting Summary linked to this article.

### Supplementary information

Reporting summary

## Data Availability

Data sharing is not applicable to this article as no datasets were generated.

## Code availability

Code availability is not applicable to this article as no custom code or scripts were used.
